# Current situation of telemedicine research for cardiovascular risk in Japan

**DOI:** 10.1038/s41440-023-01224-y

**Published:** 2023-02-27

**Authors:** Shigeru Shibata, Satoshi Hoshide

**Affiliations:** 1 Division of Nephrology, Department of Internal Medicine, Teikyo University School of Medicine, Tokyo, Japan; 2 Division of Cardiovascular Medicine, Department of Medicine, Jichi Medical University School of Medicine, Tochigi, Japan

**Keywords:** High blood pressure, Online medical counseling, Telehealth, ICT, Digital hypertension

## Abstract

Hypertension continues to be a principal risk factor for the occurrence of cardiovascular disorders, stroke, and kidney diseases. Although more than 40 million subjects suffer from hypertension in Japan, its optimal control is achieved only a subpopulation of patients, highlighting the need for novel approaches to manage this disorder. Toward the better control of blood pressure, the Japanese Society of Hypertension has developed the Future Plan, in which the application of the state-of-art information and communication technology, including web-based resources, artificial intelligence, and big data analysis, is considered as one of the promising solutions. In fact, the rapid advance of digital health technologies, as well as ongoing coronavirus disease 2019 pandemic, has triggered the structural changes in the healthcare system globally, increasing demand for the remote delivery of the medical services. Nonetheless, it is not entirely clear what evidence exists that support the widespread use of telemedicine in Japan. Here, we summarize the current status of telemedicine research, particularly in the field of hypertension and other cardiovascular risk factors. We note that there have been very few interventional studies in Japan that clearly showed the superiority or noninferiority of telemedicine over standard care, and that the methods of online consultation considerably varied among studies. Clearly, more evidence is necessary for wide implementation of telemedicine in hypertensive patients in Japan, and also those with other cardiovascular risk factors.

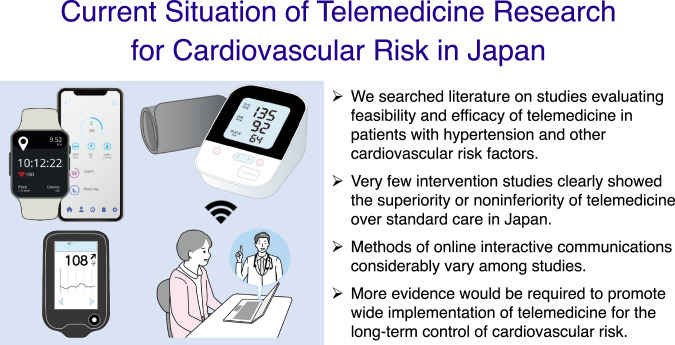

## Introduction

Hypertension affects one billion people world-wide, having a profound impact on people’s health by triggering the occurrence of cardiovascular diseases. In Japan, there are a total of 43 million subjects who live with hypertension; nonetheless, only 30% have achieved the optimal control, clearly pointing to the need for the development of distinct approaches to prevent, diagnose, and treat this disorder [1].

In an aim to improve the above situation in Japan and also to conquer hypertension, the Japanese Society of Hypertension (JSH) has developed plans for the future (JSH Future Plan) [2–4]. In that plan, the establishment of novel strategies for hypertension management by using the state-of-art information and communication technology (ICT) has been one of the key components to achieve a seamless medical care system and to facilitate the self-control of blood pressure (BP). Toward the goal, an entirely new field of research termed “digital hypertension” has also been proposed [5], in which ICT and artificial intelligence are applied to promote research and improve medical care of hypertension. It is also of note that the rapid advance of digital technologies has had a significant impact on the healthcare system globally. Moreover, the structural transition in the healthcare service has been further facilitated by the ongoing coronavirus disease 2019 (COVID-19) pandemic. However, despite the increasing need for the remote delivery of medical care, it is not entirely clear what evidence exists that support the implementation of telemedicine in Japanese patients. Globally, multiple studies and meta-analyses have been performed, which provided evidence on the efficacy of telemedicine for the management of hypertension [6–10]. Several statements on the use of telemedicine for hypertension management have also been published [11, 12]. Although those studies generally demonstrated the improved BP control compared with usual care, the methods of implementation were diverse, e.g., in terms of care givers (physician, nurse, pharmacist, or others) and ways of interaction (email, smartphone app, or online program). Moreover, duration of follow-up also varied among studies [11]. Thus, although the current pandemic has increased the need for telemedicine worldwide, there are still many challenges. With this background, we briefly summarize the current status of telemedicine research, particularly in the field of hypertension and other cardiovascular risk factors in Japan.

### Telemedicine research in hypertension

We have listed Japanese studies that have addressed the feasibility and efficacy of online medical counseling or of any kind of apps in patients with hypertension, heart failure, and other cardiovascular risk factors (Table 1). In Japan, the telemonitoring of BP has been firstly utilized for hypertensive patients who lived in a North-East area of Honshu, where the logistics and the access to medical care were severely damaged due to Great East Japan Earthquake in 2011 [13, 14]. The natural disaster could have compromised BP control and could have triggered the onset of cardiovascular disorders [15, 16]. To attenuate the risk, a web-based BP monitoring system (Disaster Cardiovascular Prevention [DCAP] network) was introduced in residents of the region [17]. Hypertensive patients received a home BP monitoring device that was connected to a web server, and local medical facilities were notified if their hypertensive patients had consistent increase in BP levels. Nishizawa et al. analyzed the BP data from the DCAP Network and reported that the average home BP of 341 hypertensive patients was decreased from 151 ± 20/87 ± 10 mmHg in May 2011 to 120 ± 12/71 ± 10 mmHg in June 2015 [14]. The study also found that the seasonal variation in BP was suppressed, showing that the use of BP telemonitoring system, along with the timely lifestyle modification and optimization of antihypertensive medication, enabled the strict BP control in these patients. These results likely indicate the attenuation of a risk of CVD events associated with seasonal BP variation [18, 19].

**Table 1:** Overview of telemedicine intervention and the use of healthcare apps for cardiovascular risk and cardiovascular disease in Japan

Study	Cardiovascular diseases or risk factors	Study setting	Sample size	Participants’ age	Study duration	Intervention	Results
Nishizawa et al., 2017 [14]	Hypertension	Prospective study	*n* = 341	71.2 years	4 years	Patients received BP telemonitoring, lifestyle modification counseling, and antihypertensive treatment.	Average home BP decreased from 151/87 mmHg to 120/71 mmHg over 4 years.
Yatabe et al., 2021 [20]	Hypertension	RCT	Standard care group (*n* = 48) Intervention group (*n* = 49)	53 years 53 years	12 months	Intervention group received web-based counseling based on home BP levels.	Average home systolic BP during the last week of the 1-year study was significantly lower in telemedicine group (125 mmHg in telemedicine group versus 131 mmHg in the standard care group; *P* = 0.02).
Kario et al., 2021 [21]	Hypertension	RCT	Standard care group (*n* = 191) Intervention group (*n* = 199)	52.0 years 52.4 years	24 weeks	Intervention group used smartphone linked system for self-monitoring (home BP) and self-management based on a personalized program of lifestyle modification.	Between-group difference in 24-hour SBP at 12 weeks as a primary endpoint was −2.4 mmHg.
Kadoya et al., 2020 [22]	Lifestyle-related diseases (including hypertension)	Retrospective study	*n* = 29	77 years	6 months	Patients received real-time interactive video-based telemedicine via a smartphone or tablet device.	No patients had exacerbation of the disease control in hypertension, dyslipidemia, and diabetes.
Kotooka et al., 2018 [28]	Heart failure	RCT	Standard care group (*n* = 90) Telemonitoring care group (*n* = 91)	65.4 years 67.1 years	15 months	Telemonitoring care group used integrated telemonitoring system consisted of body composition meter. The monitoring nurses contacted the patients by phone.	There was no statistically significant difference in all-cause death or rehospitalization of heart failure (HR, 0.95; *P* = NS).
Nagatomi et al., 2022 [29]	Chronic heart failure	RCT	Standard care group (*n* = 15) Home-based CR (*n* = 15)	67.7 years 59.8 years	3 months	Home-based CR group received online counseling via an app or by phone, based on data taken by a smartphone-linked wearable device, symptoms, BP, BW, etc.	Home-based CR group showed significant improvement in the 6-min walking distance compared with standard care group.
Saitoh et al., 2022 [30]	Cardiac disease	RCT	Standard care group (*n* = 5) Intervention group (*n* = 6)	68 years 74 years	3 months	Intervention group used tablet linked system for self-monitoring and management of physical (symptom, BP, BW), exercise and nutrition.	There was no difference in the change in the functional status and short-term prognosis between groups.
Kikuchi et al., 2021 [31]	Heart failure	Prospective single arm study	*n* = 10	76 years	12 weeks	Participants used integrated telerehabilitation platform consisted of IoT-equipped ergometer and ECG monitoring.	The system was feasible and safe. Six-minute walk distance significantly improved from 383 m to 432 m (*P* = 0.003).
Nakayama et al., 2020 [48]	Heart failure	Retrospective non-randomized	Outpatient CR (*n* = 69) Remote CR (*n* = 30) Non-CR (*n* = 137)	59 years 70 years 69 years	30 days	Patients in remote CR received telephone call by a doctor or nurses biweekly to check symptoms, vital signs, and activity.	The emergency readmission rate within 30 days of discharge was lower in the remote CR group than in the non-CR group (0% vs. 3%, *P* = 0.02).
Onishi et al., 2022 [39]	Diabetes	Retrospective study	*n* = 2727	68.6 years	About 5 months (Pre and post emergency period in 2020)	Patients received telephone call by doctor instead of office visit. They were advised to check their bodyweight, not to overeat, to exercise and not gain weight while they stayed home. The frequency and methods of telemonitoring were determined by each doctors.	Both office visit (OR, 1.53) and telemedicine (OR, 1.56) were independently associated with the HbA1c levels of < 7.0%.
Onishi et al., 2022 [38]	Diabetes	Retrospective study	Pre-period of COVID-19, 2019 years (*n* = 3608) Post-period of COVID-19, 2020 years (*n* = 3333)	68.5 years 68.7 years	-	Same as above.	In year 2020, office visit group had a significantly better post-HbA1c levels than telemedicine group.
Yamaguchi et al., 2019 [49]	Diabetes	Observational study	*n* = 522	40–59 years	1 year	Participants used an app that integrates the information on blood glucose, body weight, daily steps, and diet.	Robust users (≥ 4 weeks) had higher daily step counts compared to non-robust users (6108 vs. 5171; *P* = 0.001).
Nomura et al., 2019 [35]	Smoking	RCT	Standard care group (*n* = 57) Telemedicine group (*n* = 58)	53 years 55 years	24 weeks	Telemedicine group used an app for smoking cessation.	Both telemedicine and control groups had similar continuous abstinence rate from weeks 9 to 12 (81.0% vs 78.9%) and the lower limit of the difference between groups (–12.8%) was greater than the prespecified limit (–15%).
Kato et al., 2020 [36]	Smoking	Prospective single arm study	*n* = 177	44.6 years	24 weeks	Participants used an app for smoking cessation.	Continuous abstinence rates were 48.6% and 47.5% at 9–12 weeks and 21–24 weeks, respectively.
Masaki et al., 2019 [37]	Smoking	Prospective single arm study	*n* = 55	43.3 years	24 weeks	Participants used an app for smoking cessation.	Continuous abstinence rate from weeks 9 to 24 was 64%.
Murase et al., 2020 [43]	Sleep apnea	RCT	Telemedicine group (*n* = 161) Face to face 3 months follow group (*n* = 166) Face to face 1 month follow group (*n* = 156)	60 ± 11 years 60 ± 13 years 61 ± 12 years	6 month	In telemedicine group, physicians checked CPAP adherence data monthly using a telemonitoring system and called patients to improve adherence, in addition to office visits every 3 months.	CPAP adherence in telemedicine group was not inferior to monthly face-to-face follow-up.
Kondo et al., 2022 [50]	Obesity	RCT	Standard care group (*n* = 34) Intervention group (*n* = 41)	48.5 years 49.3 years	3 months	Intervention group used smartphone-linked system for self-monitoring and management of physical (BP, BW, BG), exercise and nutrition.	The average change of visceral fat area was −23.5 cm^2^ in the intervention group and 1.9 cm^2^ in the control group.
Hamaya et al. 2021 [51]	Health check-up population	Observational study	*n* = 12,602	44.1 years	12 months	An app-linked annual health check-up data, daily pedometer, and insurance claim data were analyzed.	Those in the highest quintile in daily step change had, compared with those of the lowest quartile, a significant reduction in weight (–0.92 kg, *P* < 0.001), LDL cholesterol (–2.78 mg/dL, *P* = 0.004), and HbA1c (–0.04%, *P* = 0.004), and increase in HDL cholesterol (+1.91 mg/dL, *P* < 0 .001).
Hayashi et al., 2017 [52]	Dialysis patient	Prospective single arm study	*n* = 9	47.9 years	16.9 weeks	Participants used an app that displayed interdialytic weight gain, serum potassium, and serum phosphate.	The average completion rates were 78 to 95%. Of seven participants who completed the usability survey, six reported that the app helped improve the self-management.

Data are the number, median, median, or range

*BP* blood pressure, *BW* body weight, *CPAP* continuous positive airway pressure, *CR* cardiac rehabilitation, *HR* hazard ratio, *MWD* minute walk distance, *RCT* randomized control trial

There are several other studies that assessed the efficacy of telemedicine in hypertensive patients. Yatabe et al. recruited 99 patients with uncomplicated hypertension and randomized them to either telemedicine group or standard care group [20]. All the patients were instructed to measure BP twice every morning (before having a breakfast) and every evening (before going to bed) by using a uniform, validated automatic sphygmomanometer. In the telemedicine group, the measured BP was transmitted to physicians through a mobile communication system, and the patients received prescription after web-based video visit every 6 weeks for 1 year. In the standard care group, BP was monitored using the same device but was managed through the office visits. BP levels were similar between two groups at baseline (average BP for all participants, 136 ± 13/91 ± 9 mmHg). The primary outcome of the study, which was the average home systolic BP during the last week of the 1-year study, was significantly lower in telemedicine group (125 ± 9 mmHg in telemedicine group versus 131 ± 12 mmHg in the standard care group; *P* = 0.02).

In a prospective, open-label, randomized controlled trial, Kario et al. evaluated the efficacy of an app-supported lifestyle modification in BP control [21]. In this study, 390 patients with essential hypertension who had not used antihypertensive medication for ≥ 3 months were assigned to digital therapeutics group or control group. All the study participants were provided with a detailed instruction on lifestyle modifications recommended by the JSH and the patients in digital therapeutics group received additional interactive support for lifestyle modification by the app that retrieves data from home BP monitoring device. BP data, as well as daily activity information, were simultaneously transferred to healthcare providers. The primary efficacy endpoint, which was the mean change in 24 h ambulatory systolic BP from baseline to 12 weeks, were −4.9 mmHg in digital therapeutics group as compared with −2.5 mmHg in control group (between-group difference, −2.4 mmHg; 95% CI, −4.5 to −0.3 mmHg; *P* = 0.02). Home and office systolic BP was also significantly lower in the digital therapeutics group [21].

In a prospective observational study, Kadoya et al. enrolled 34 patients with non-communicable diseases including hypertension, dyslipidemia, and diabetes mellitus [22]. In the recommended study protocol, telemedicine consultations were scheduled at 1, 2, 4, and 5 months; office visit was scheduled at 3 and 6 month to comply with the regulation of the National Health Insurance System in Japan. In the telemedicine visit, patients received real-time video-based consultation via a smartphone or a tablet. After 6 months, five subjects were excluded from the study because they did not or were not able to receive telemedicine consultations. Among 24 patients with hypertension who completed the study, average BP was 138/71 mmHg at baseline and 140/75 mmHg at 6 month (*P* = 0.57). Levels of HbA1c and lipids also remained unchanged in the study participants at 6 months. In sum, telemonitoring of BP mainly based on home BP measurement is feasible and seems to contribute to improved BP control. Given that there is a number of evidence showing the association between home BP and CVD risk [23–26], it is no doubt that home BP measurement will continue to play a central role in telemedicine for hypertension. Of course, its efficacy in a long term merits further investigation.

### Telemedicine research in heart failure and cardiac rehabilitation

Remote monitoring has been proposed to be a potentially useful approach to improve the outcome of patients with chronic heart failure [27]. There are several studies in Japan that addressed telemonitoring and home cardiac rehabilitation (CR) in patients with heart failure. In HOMES-HF [28], which was a multicenter, open-label, randomized, controlled trial, a total of 181 recently hospitalized patients with New York Heart Association (NYHA) class II-III heart failure were assigned to telemonitoring group or usual care group. A home telemonitoring system used in that study received the data on BP, pulse rate, body weight and body composition and transmitted to a central web server, which was monitored by full-time nurses at a medical facility. The nurses then notified the patient’s physician if the acquired data exceeded the threshold that had been prespecified for each patients. The mean rate of adherence to the telemonitoring was 90.9% at 12 months, showing the feasibility of the approach. However, the primary endpoint, which was a composite of all-cause death or re-hospitalization due to worsening heart failure, was not significantly different between groups (hazard ratio, 0.95; 9% CI 0.55-1.65).

Nagatomi et al. evaluated whether home-based cardiac rehabilitation (CR) combined with online medical counseling improved the physical activity in patients with chronic heart failure compared with the standard care [29]. In that single-center, open-label, randomized, controlled trial that included 30 patients with NYHA II-III heart failure, subjects were assigned to home-based CR group or control group. In the home-based CR group, participants used a wearable device that was able to transfer the data such as heart rate and daily activity to the medical facility via a smartphone app. The training menu consisted of three to five times a week of aerobic exercise and two to three times a week of resistance training. Home-based CR team composed of physical therapists, dietitians, nurses, and cardiologists communicated approximately once a week via a messaging tool of the app or by phone to plan the training frequency and intensity in coming weeks. The control group received standard pharmacological and non-pharmacological treatment but not home-based CR. The primary outcome, which was the change in the 6-min walking distance at 3 months, was 52.1 ± 43.9 m in the home-based CR group and −4.3 ± 38.8 m in the control group; the observed changes in the walking distance was significantly greater in home-based CR group (*P* < 0.001). Feasibility of remote CR was also addressed in a study by Saitoh et al. In that randomized controlled study, 11 patients received either center-based CR or remote CR after completion of 12-week ambulatory CR program [30]. In the remote CR group, the patients’ data (BP, body weight, heart rate, electrocardiogram, oxygen saturation, medication adherence, and physical activity) were recorded in a telemedicine system connected to a tablet. Based on data, physical therapists provided feedback once a week. In the center-based CR group, the patients received once or twice a week of CR at the hospital for 4 weeks. The authors found no difference in functional status between groups after the 4-week follow-up.

Kikuchi et al. evaluated the efficacy of remote monitoring system for home-based CR [31]. In that prospective, single-arm study, 10 patients with heart failure who were not able to participate in outpatient CR program were included. The home-based CR consisted of three sessions per week for 12 weeks. The tele-rehabilitation system used in the study was composed of ergometer and electrocardiography connected to a tablet, which allowed real-time supervision of CR sessions from the medical facility. The median participation rate was 94.4%; although several patients reported fatigue and palpitations, no serious cardiovascular events were reported. Six-minute walk distance and low extremity muscle strength were improved after 12 weeks.

Although several guidelines recommend CR for the secondary prevention of not only heart failure but also ischemic heart disease [32, 33], a previous randomized controlled study demonstrated that there was no significant reduction in all-cause mortality or hospitalization in patients with chronic heart failure assigned to exercise training group [34]. In that study, supervised sessions for 3 months were followed by home-based exercise for the rest of the period during the median follow-up of 30.1 months [34]. It would be possible that the application of telemedicine increases the benefit of home-based training by improving adherence and safety.

### Telemedicine in other cardiovascular risk factors

Several studies have addressed the efficacy of telemedicine in controlling cardiovascular risk factors such as smoking, diabetes mellitus, and sleep apnea. To determine whether the application of telemedicine is effective in smoking cessation, Nomura et al. randomized 115 nicotine-dependent smokers with a Brinkman index of ≥200 to receive either internet-based video counseling (telemedicine arm) or standard smoking cessation program (control arm) in Japan [35]. All the participants were provided with a smartphone app equipped with a mobile carbon oxide checker. Instead of visiting clinics, those allocated to the telemedicine arm met their physicians via internet. Both groups had similar continuous abstinence rate from weeks 9 to 12 (telemedicine arm, 81%; 95% CI, 71 to 91%; control arm, 79%; 95% CI, 68 to 89%). The absolute difference between two group was 2.1% (95% CI, −12.8 to 17.0%), which was greater than prespecified noninferiority margin of −15%. The study concluded that the efficacy of telemedicine-based smoking cessation program was noninferior to that of the office-visit-based program [35].

Kato et al. retrospectively investigated data of 177 smokers who received online counseling combined with digital therapeutics for 24 weeks to quit smoking [36]. Online sessions with professional nurses or pharmacists were provided at 1, 2, 4, 8, 12, and 24 weeks. In that study, program adherence rate was 72% (127/177) at 12 weeks and 60% (106/177) at 24 weeks; continuous abstinence rates were 49% (86/177) and 47% (84/177) at weeks 9–12 and weeks 21–24, respectively. Given the report showing that the completion rate of 12-week smoking cessation program in Japan was around 30%, the authors concluded that the program showed favorable continuous abstinence rates [36]. In a prospective, single-arm study, Masaki et al. evaluated the efficacy of the same smoking cessation app in 55 subjects [37]. In that study, participants visited clinics at 0, 2, 4, 8, 12 weeks. Physicians were also able to monitor patients’ data uploaded to a server. The study reported that the continuous abstinence rates were 64% at 9 to 24 weeks, 76% at 9 to 12 weeks, and 58% at 9 to 52 weeks.

Onishi et al. retrospectively evaluated the efficacy of telemedicine and clinic visit on glycated hemoglobin (HbA1c) levels during the state of emergency in response to the outbreak of COVID-19 [38, 39]. Telemedicine consisted of telephone consultation and its application was determined by the doctors based on patients’ health status, living areas, and other conditions. In a multiple regression analysis that involved 2727 diabetic patients, both telemedicine visits and office visits were independently associated with the improved HbA1c levels (<7%) evaluated 8 weeks after the state of emergency [39]. However, in the propensity score analysis that involved 618 pairs with pre-HbA1c levels of 7% or higher, the clinic visit group had a significantly better post-HbA1c levels than the telemedicine group (7.4% vs. 7.5%; *P* = 0.02) [38].

Continuous positive airway pressure (CPAP) has been established for the treatment of obstructive sleep apnea (OSA). However, previous studies reported that CPAP treatment did not result in significant reduction in CVD events compared to non-CPAP treatment in OSA patients [40–42]. On the other hand, the results of those studies may also indicate the importance of adherence to CPAP treatment. One multicenter study conducted in 17 sleep centers across Japan evaluated the noninferiority (margin set at 15%) of telemedicine visit on adherence to CPAP treatment compared with face-to-face visit in 483 patients with sleep apnea [43]. In that prospective randomized trial, patients who had used CPAP for >3 months were assigned to a monthly telemedicine group, a group of 3-month interval in-person visit (3-month group) or a group of 1-month interval in-person visit (1-month group). Patients with telemedicine group visited the clinic every 3 months; in the months without the clinic visit, physicians checked the CPAP adherence data that were transferred via wireless modems attached to CPAP machines. If the days with ≥4 h/night use of CPAP was less than 70% in that month, physicians then called their patients to improve the adherence. During the study, 32.3% of patients with telemedicine group received telephone coaching from the physicians. Decreased adherence (defined as the decline of ≥5% in CPAP use from baseline) was found in 25.5% of the participants in telemedicine group, 33.1% in 3-month group, and 22.4% in 1 month-group at the end of 6-month study period. Comparison among groups demonstrated that telemedicine group was noninferior to 1-month group (difference between the two groups was 3.0%; 95% CI, −4.8% to 10.9%; *P* < 0.01), whereas 3-month group did not show noninferiority to 1-month group (10.7%; 95% CI, 2.6 to 18.8%; *P* = 0.19). Thus, telemedicine support could help improve the adherence to CPAP use in patients with sleep apnea. Good CPAP adherence has been negatively associated with morning home BP on the following day [44], which would result in the reduction in CVD events related to day-by-day home BP variability [45–47].

### Summary and perspectives

We have summarized the current status of telemedicine research in Japan, with an emphasis on hypertension-related disorders and risk factors. As noted above, there were very few intervention studies in Japan that clearly showed the superiority or noninferiority of telemedicine over standard care. The methods and frequencies of the online interactive communications between patients and healthcare providers also varied among studies. If the purpose of telemedicine is to reduce office visits and person-to-person contacts, as is the case in COVID-19 pandemic and natural disasters, it might not be necessary to prove the superiority of telemedicine over the conventional treatment. Indeed, usefulness of telemedicine is well recognized in these special occasions [13]. Nonetheless, it would be still required to establish a common approach to implement telemedicine, such as frequency and methods of online counseling. In addition, given that the current medical evidence is mostly based on the basis of in-person medical counseling in a usual state, there is also a need to develop a sort of guidelines for the implementation of telemedicine to control and improve the quality. For example, one study found that HbA1c levels in diabetic patients were rather worse in the telemonitoring group [38]; in that study, the application of telemedicine depended on each physician’s decision.

The studies summarized here demonstrated that telemedicine was overall feasible. However, all but one study followed the patients for a year or less; the long-term consequence of the application of telemedicine remains unclear. It is particularly important for those with cardiovascular risk factors, including hypertension, to determine the feasibility and efficacy in a longer period, ideally 10 years or more. For certain disorders, elderly people would most benefit from a telemedicine system, e.g., those with chronic heart failure. However, it is generally difficult for the elderly to use a telemedicine system with a smartphone application. Actually, participants in the studies that used smartphone were relatively young compared with those in the studies that did not use smartphone. It also needs to note that the devices and apps are advancing so rapidly that it may be difficult to provide a timely evidence on CVD prevention. Still, current evidence seems insufficient and more data are required for the wide implementation of telemedicine in managing cardiovascular risk factors, such as hypertension.
